# Cerebellar tDCS and pain modulation: a critical integrative and systematic review

**DOI:** 10.3389/fneur.2025.1681853

**Published:** 2025-10-31

**Authors:** Daniel Fernando Arias Betancur, Maria da Graça Lopes Tarragó, Maria Eduarda Louzada Oliveira, Sara Machado Peres, Iraci L. S. Torres, Felipe Fregni, Wolnei Caumo

**Affiliations:** ^1^Graduate Program in Medical Sciences, School of Medicine, Federal University of Rio Grande do Sul (UFRGS), Porto Alegre, Brazil; ^2^Laboratory of Pain & Neuromodulation, Clinical Research Center, Hospital de Clínicas de Porto Alegre (HCPA), Porto Alegre, Brazil; ^3^Physical Medicine and Rehabilitation Service, Hospital de Clínicas de Porto Alegre (HCPA), Porto Alegre, Brazil; ^4^Pharmacology of Pain and Neuromodulation: Pre-clinical Investigations Research Group, Federal University of Rio Grande Do Sul (UFRGS), Porto Alegre, Brazil; ^5^Laboratory of Neuromodulation and Center for Clinical Research Learning, Physics and Rehabilitation Department, Spaulding Rehabilitation Hospital, Boston, MA, United States; ^6^Pain and Palliative Care Service, Hospital de Clínicas de Porto Alegre (HCPA), Porto Alegre, Brazil; ^7^Department of Surgery, School of Medicine, Federal University of Rio Grande do Sul (UFRGS), Porto Alegre, Brazil

**Keywords:** cerebellar tDCS, pain modulation, chronic pain, neuromodulation, systematic review

## Abstract

**Background:**

Cerebellar transcranial direct current stimulation (ctDCS) has emerged as a promising non-invasive neuromodulatory approach for managing pain. Early evidence suggests beneficial effects on pain perception in both healthy individuals and patients with chronic pain. However, the underlying mechanisms and clinical efficacy remain unclear. This systematic review aimed to synthesize the current evidence on cerebellar involvement in pain processing and to evaluate the potential of ctDCS as a therapeutic intervention.

**Methods:**

A systematic search was conducted in PubMed, Embase, and the Cochrane Library, following PRISMA guidelines. MeSH and Emtree descriptors related to “Cerebellum,” “Pain,” and “tDCS” were used to identify relevant studies published up to December 11, 2024. Eligible studies were randomized controlled trials (RCTs) that investigated the effects of ctDCS on pain. Risk of bias was assessed using the Cochrane Risk of Bias Tool version 2 (RoB 2).

**Results:**

Of 819 records screened, five RCTs met the inclusion criteria. The primary methodological limitations included incomplete reporting of randomization procedures and inadequate blinding of outcome assessors. Two studies lacked key demographic and clinical details, while one showed a high risk of bias due to repeated same-day stimulation. Despite these issues, Across the included studies anodal ctDCS generally increased pain thresholds and enhanced endogenous pain inhibition, whereas cathodal ctDCS tended to reduce thresholds. Neurophysiological findings supported these behavioral results, with EEG data showing modulation of cortical activity related to pain processing.

**Conclusion:**

Preliminary findings suggest that ctDCS may modulate nociceptive pathways and enhance pain inhibition. However, the small number of studies and methodological heterogeneity limit the generalizability of current results. Further high-quality RCTs are needed to optimize stimulation protocols, assess long-term effects, and establish clinical benefits. This review supports the cerebellum as a relevant and underexplored target for neuromodulatory pain interventions.

## 1 Introduction

Chronic pain affects approximately 20% of the adult population in Western countries and represents a major public health challenge due to its multifactorial nature and significant impact on quality of life (1). Typically, it is defined as pain occurring on most days or every day for more than three months (2). Despite advances in both pharmacological and non-pharmacological approaches, effective management remains elusive. Recent U.S. data show that 24.3% of adults reported chronic pain in 2023, and 8.5% experienced high-impact chronic pain—pain that frequently limits daily or work activities—reflecting a worsening trend and emphasizing the need for more personalized and integrated therapeutic approaches (3–6).

In response to the growing burden of chronic pain, there has been renewed interest in innovative treatments targeting central pain modulation mechanisms. Non-invasive neuromodulation techniques have shown promise in enhancing pain outcomes. Among these, transcranial direct current stimulation (tDCS) has emerged as a compelling modality, demonstrating efficacy across a range of pain conditions, coupled with a favorable safety profile and low incidence of adverse effects (7, 8).

Most neuromodulation research to date has focused on cortical targets, particularly the dorsolateral prefrontal cortex (DLPFC) (9, 10) and the primary motor cortex (M1) (10, 11). However, increasing attention has recently turned to the cerebellum as a novel target for non-invasive stimulation. While traditionally associated with sensorimotor integration, the cerebellum is now recognized as a key modulator of pain through its role in integrating nociceptive input with motor and cognitive-affective processes, thereby influencing both pain perception and emotional reactivity (12).

Functional neuroimaging studies have further elucidated the cerebellum’s role in pain processing, revealing its interactions with key regions, including the anterior midcingulate cortex, supplementary motor area, and thalamus (13, 14). Although emerging studies involving both healthy individuals and patients with neuropathic pain offer promising insights (15–19), several critical gaps remain. These include uncertainties regarding optimal stimulation parameters, the precise mechanisms of action, long-term efficacy, and the potential benefits of multisite stimulation protocols targeting regions such as M1 and DLPFC.

To advance the field, this review offers a comprehensive analysis of cerebellar involvement in pain processing, drawing on anatomical, clinical, and experimental evidence. It explores the role of the cerebellum in pain modulation and evaluates the therapeutic potential of ctDCS. Key technical considerations, current limitations, and future directions for research and clinical application are also discussed.

## 2 Mapping cerebellar circuits: anatomical insights into pain processing

### 2.1 Cerebrocortical and cerebellar contributions to pain modulation

Pain is a multidimensional experience arising from the interplay of sensory, affective, cognitive, and social factors (20), processed through distributed brain networks that include the thalamus, brainstem nuclei, somatosensory cortices, and prefrontal regions (21). Among cortical targets, the M1 and DLPFC have been the most extensively investigated in neuromodulation. Stimulation of M1 enhances excitability within intracortical circuits, thalamic relays, and descending modulatory pathways, producing analgesic effects partly mediated by endogenous opioids (22). The DLPFC, in turn, contributes to top-down regulation of cognitive-affective processes, strengthening connectivity with the periaqueductal gray (PAG) and engaging in expectation-driven analgesia (23). Evidence from clinical and preclinical studies shows that modulation of these regions not only reduces pain intensity but also alleviates maladaptive cognitive-emotional factors such as catastrophizing and depressive symptoms (24–26).

Beyond these well-established cortical sites, increasing evidence highlights the cerebellum as a promising and integrative hub in pain processing. Far from being solely a motor structure, the cerebellum contributes to sensory, cognitive, and emotional dimensions of the pain experience (27, 28). Its activation has been reported across various pain states, including acute, chronic, and neuropathic pain (21, 29). Together, these findings position the cerebellum as a novel neuromodulatory target, with ctDCS offering a promising approach to influence distributed pain-related networks through cerebellar stimulation.

### 2.2 Anatomical and functional framework of the cerebellum in pain

The cerebellum constitutes only a small fraction of total brain volume, yet it contains more than half of all neurons in the central nervous system (27, 30). Traditionally regarded as a motor structure, converging evidence highlights its integrative role in cognitive and emotional processing (31, 32). Structurally, it comprises the vermis, paravermal zones, and lateral hemispheres, which form the anterior, posterior, and flocculonodular lobes (33). Functionally, it is classically divided into the vestibulocerebellum (balance and oculomotor control), spinocerebellum (muscle tone and proprioception), and cerebrocerebellum (motor planning and execution) (34).

At the microcircuit level, mossy and climbing fibers provide the main excitatory inputs to the cerebellar cortex, converging on Purkinje cells that exert inhibitory control over the deep cerebellar nuclei. These nuclei—fastigial, dentate, globose, and emboliform—constitute the principal output hubs, projecting to motor, sensory, limbic, and associative regions, thereby linking the cerebellum to both motor and non-motor functions (35–38). This highly organized architecture provides the substrate for cerebellar contributions to pain processing, enabling integration of sensory, affective, and cognitive information.

**Figure 1:**
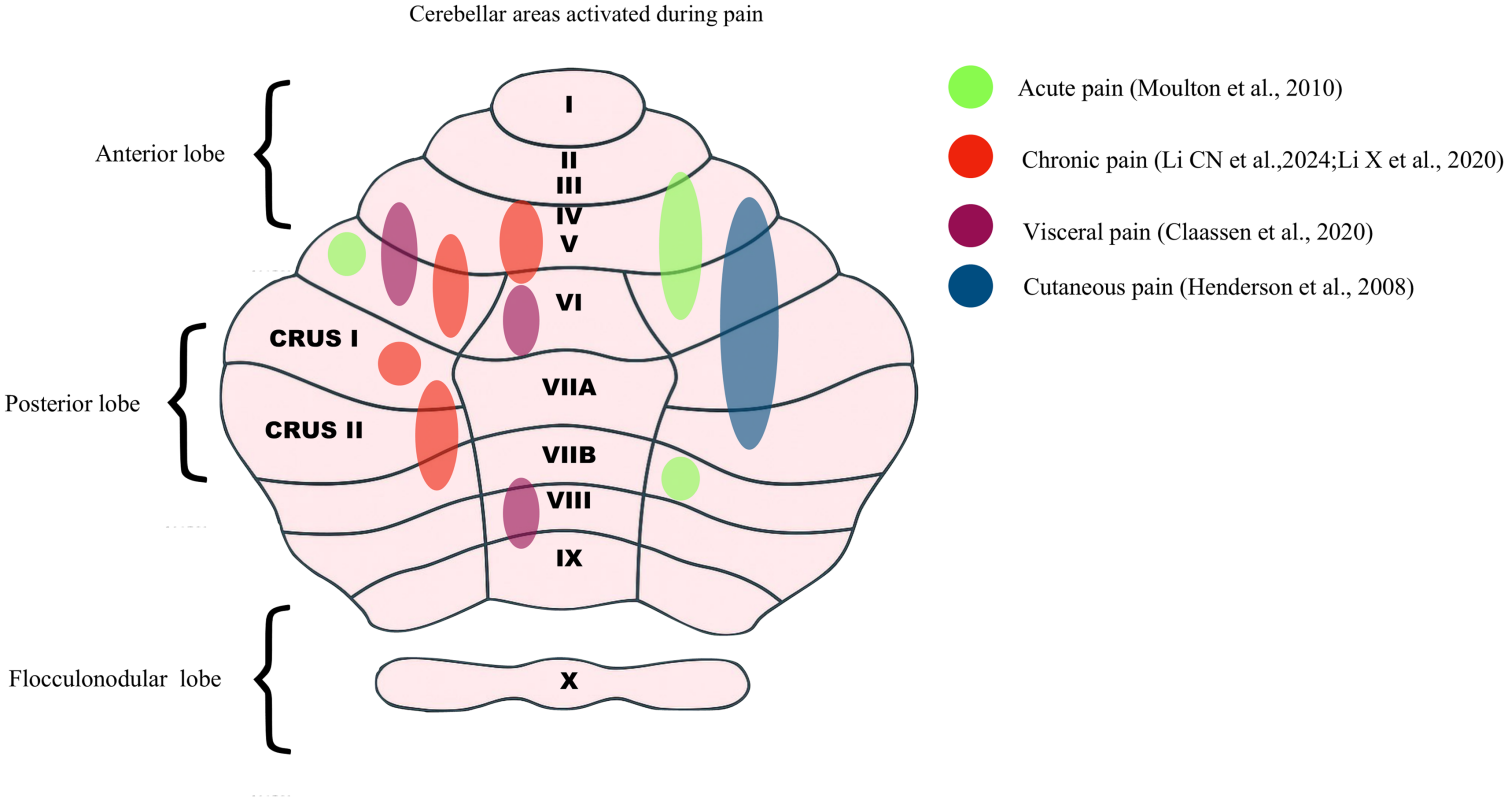
Cerebellar activation patterns in different pain states. The diagram illustrates brain regions activated during acute pain (lobules III–VI, VIIb, Crus II), chronic pain (vermal IV–V, hemispheric V–VI, Crus I), visceral pain (bilateral V–VI, Crus I, VIII, vermal VI), and somatic pain (lateral cerebellar cortex with sex-related differences).

Topographical mapping further reveals pain-related specialization. Larsell’s classification divides the cerebellum into ten lobules (I–X), which display functional heterogeneity. Sensorimotor processing predominates in lobules I–IV and VIII (39), whereas lobules VI–IX support emotional and cognitive regulation (32), and lobules V–VII and IX–X contribute to vestibular and interoceptive integration (40, 41). Neuroimaging studies show that acute pain recruits lobules III–VI, VIIb, Crus II, and bilateral hemisphere VI (28), whereas chronic pain preferentially engages vermal lobules IV–V and hemispheric lobules V, VI, and Crus I—patterns consistent with sustained pain states and emotional dysregulation (28, 42). Distinct networks have also been described in visceral pain, with consistent activation of bilateral lobules V–VI, Crus I, lobule VIII, and vermal VI (43). In the context of somatic pain, sex-related differences have been observed: men exhibit increased activation of the lateral cerebellar cortex following muscle and cutaneous stimulation, whereas women display reduced or absent responses; this pattern may reflect variability in endogenous analgesic systems (44). Beyond the direct encoding of pain, anticipatory mechanisms have also been identified, with ipsilateral posterior cerebellar activation preceding noxious heat stimulation, suggesting a role in sensory prediction and pre-attentive processing (45). Collectively, these findings highlight the heterogeneity of cerebellar activation across pain modalities, as summarized in Figure 1.

Taken together, these anatomical and functional features position the cerebellum as a dynamic hub for integrating sensorimotor, autonomic, and affective information in pain processing. This organizational scaffold forms the basis for the afferent and efferent cerebrocerebellar circuits described in the following sections.

### 2.3 Cerebrocerebellar afferent systems involved in pain processing

The cerebellum receives afferent input from diverse cerebral sources involved in motor, somatosensory, cognitive, affective, and reward processing. The convergence of these signals allows cerebellar circuits to integrate nociceptive information and modulate pain perception across multiple functional domains.

Excitatory input reaches the cerebellar cortex through two principal pathways. Mossy fibers, originating from various brainstem nuclei, form excitatory synapses with granule cells and golgi cells in the granular layer. The axons of these granule cells ascend into the molecular layer and bifurcate into parallel fibers that synapse with the dendrites of Purkinje cells, thereby activating them. This activation ultimately produces inhibitory output from Purkinje cells to the deep cerebellar nuclei, contributing to both motor and non-motor adaptive responses (33, 46).

**Figure 2:**
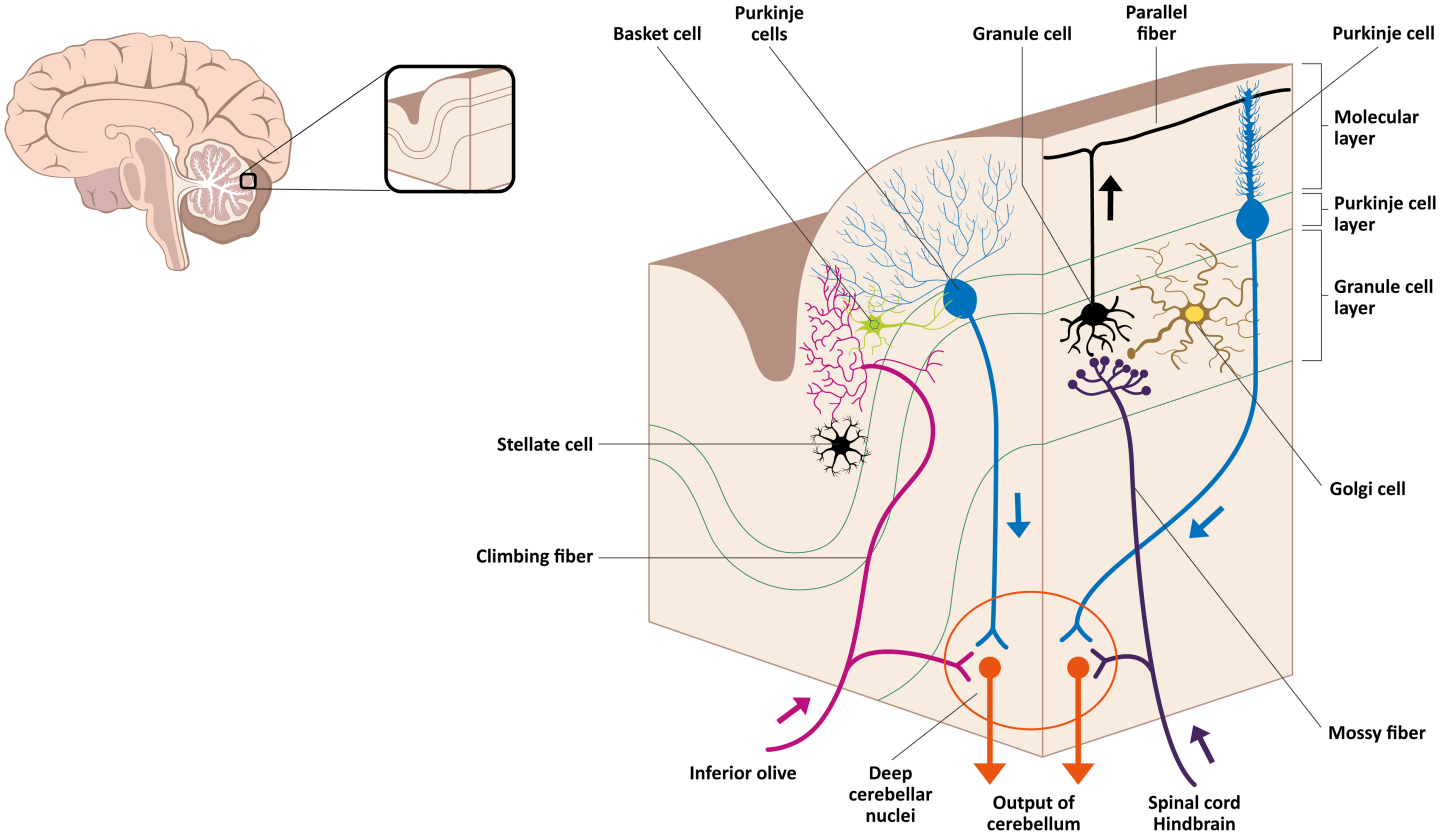
Afferent convergence in the cerebellar cortex and deep nuclei. Mossy fibers from brainstem and spinal relays excite granule and Golgi cells in the granular layer; granule axons ascend as parallel fibers and contact Purkinje dendrites in the molecular layer. Climbing fibers from the inferior olive form powerful synapses on Purkinje dendrites and send collaterals to the deep cerebellar nuclei. The inhibitory output of Purkinje neurons to these nuclei, which relay to thalamic and brainstem targets, provides an anatomical substrate consistent with the integration of nociceptive, sensorimotor, and affective information.

In parallel, climbing fibers arising from the contralateral inferior olivary nucleus establish powerful excitatory synapses with Purkinje cells, inducing long-term depression (LTD) at parallel fiber–Purkinje cell synapses (47). These fibers also send collateral projections to the deep cerebellar nuclei and engage inhibitory interneurons, including basket and stellate cells, which promote lateral inhibition of neighboring Purkinje cells (48, 49). Through these mechanisms, climbing fibers convey precise timing and prediction-error signals essential for sensorimotor integration and pain modulation (50, 51). These connections between mossy- and climbing-fiber inputs and cerebellar cortical and nuclear elements are illustrated in the Figure 2.

Convergent nociceptive-related signals reach cerebellar circuits via both cerebrocerebellar and spino-olivocerebellar pathways. Among the most prominent are the corticopontocerebellar projections, which originate from M1, premotor cortices, DLPFC, and primary somatosensory cortex (S1), and relay through the pontine nuclei to reach the cerebellar cortex (52–56). Projections from M1 and S1 primarily target lobules I–V and VI, delivering motor efference copies and somatosensory feedback critical for fast adaptive responses during nociceptive events (57). The lateral hemisphere, especially Crus I, receives input from the DLPFC and parietal cortices, providing a substrate for integrating attention, salience, and pain expectation (50, 58). In addition, dopaminergic projections from the ventral tegmental area (VTA) terminate predominantly in Crus II and, to a lesser extent, in Crus I. This mesolimbic-cerebellar pathway forms a neuroanatomical interface between motivational-affective processing and cerebellar circuits, implicating the cerebellum in reinforcement learning and the drive for analgesia (59–61).

**Figure 3:**
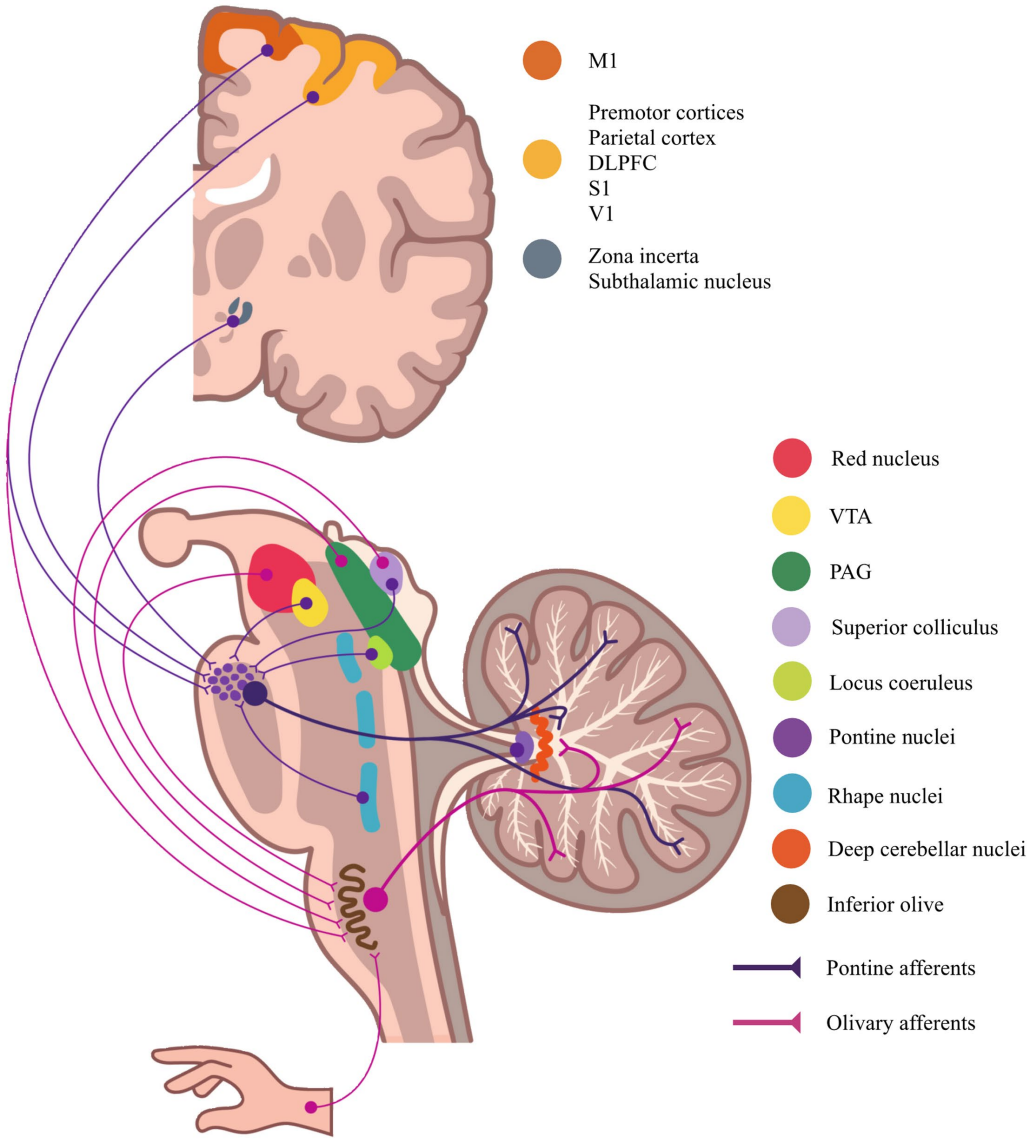
Topographical mapping of afferent projections to the cerebellum from brain regions involved in motor, sensory, cognitive, affective, and reward processing. M1 – primary motor cortex; S1 – primary somatosensory cortex; DLPFC – dorsolateral prefrontal cortex; V1 – visual cortex; PAG – periaqueductal gray; VTA – ventral tegmental area. This schematic illustrates the multidimensional integration of nociceptive inputs by the cerebellum through mossy and climbing fibers, which contributes to the modulation of sensory-discriminative, affective, and cognitive components of pain.

Complementing these cerebrocortical inputs, animal studies have shown that direct nociceptive information can reach the cerebellum via the spino-olivocerebellar pathway. Ekerot et al. (60) demonstrated that stimulation of A- and B-type cutaneous nociceptive fibers in animal models triggered climbing fiber-mediated activation of Purkinje cells in the anterior cerebellum. This effect was likely mediated by the spino-olivocerebellar tract, which ascends via the dorsal and dorsolateral funiculi to deliver nociceptive input directly to cerebellar circuits (28, 62). The inferior olive itself receives convergent input from midbrain structures involved in pain control, including the red nucleus, zona incerta, and the periaqueductal gray (PAG), a key hub of descending pain modulation (63). Figure 3 schematically illustrates the topographical and anatomical organization of these afferent systems.

### 2.4 Cerebrocerebellar efferent pathways modulating nociception

**Figure 4:**
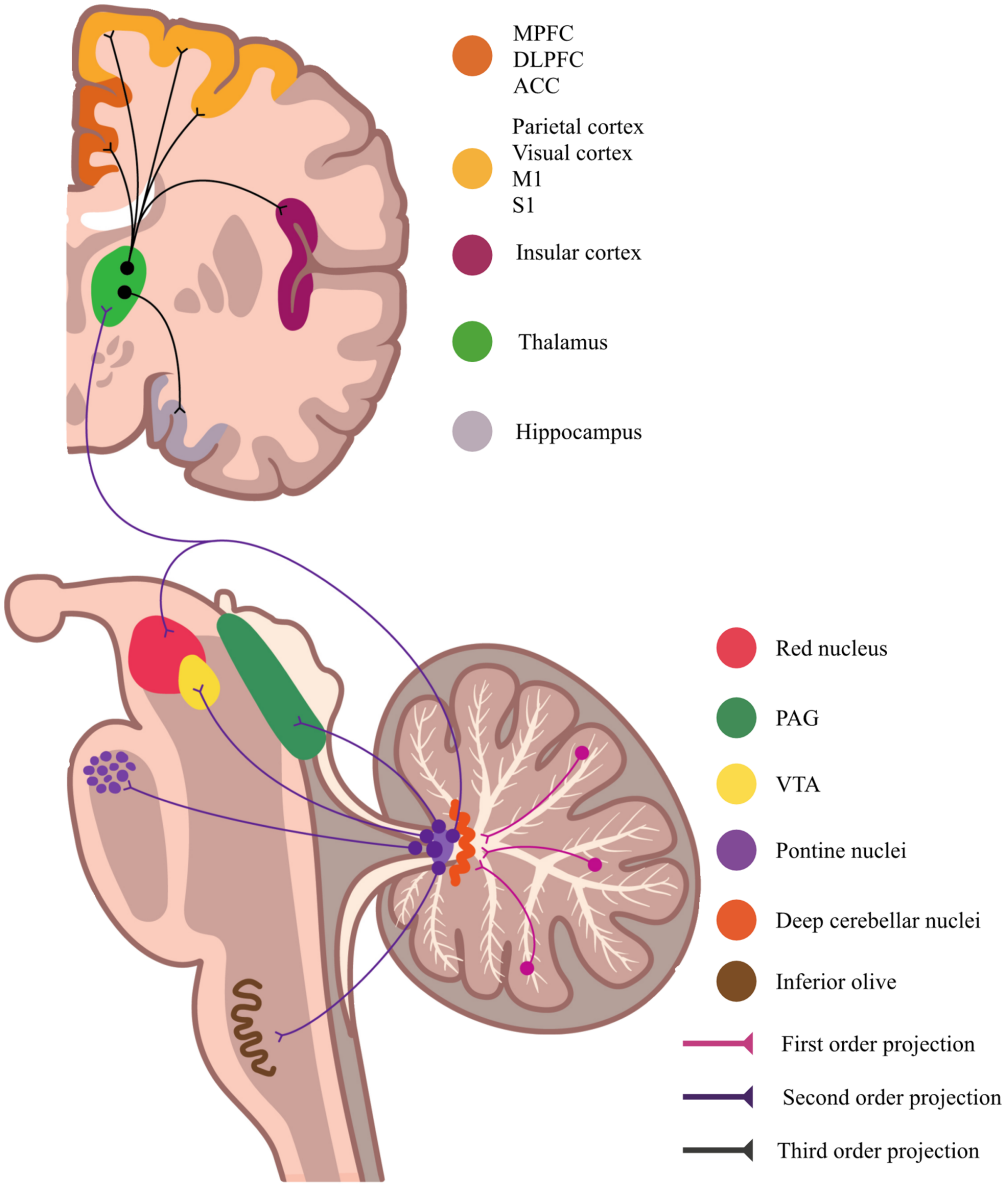
Efferent cerebellar pathway potentially involved in pain modulation. Schematic illustration of the dentatothalamocortical tract, an efferent pathway projecting from the dentate nucleus of the cerebellum to the thalamus, and subsequently to cortical regions such as the primary motor cortex and prefrontal cortex. This tract is thought to contribute to the modulation of both sensorimotor and affective-cognitive components of pain processing. Abbreviations: M1 – primary motor cortex; S1 – primary somatosensory cortex; DLPFC – dorsolateral prefrontal cortex; MPFC – medial prefrontal cortex; ACC – anterior cingulate cortex; PAG – periaqueductal gray; VTA – ventral tegmental area.

Among cerebellar efferent pathways, projections originating in lobules IV–VI and Crus I/II provide the structural substrate for top-down modulation of pain-related behaviors. Purkinje cells in these lobules send inhibitory outputs to the deep cerebellar nuclei, which in turn project excitatory fibers to the motor thalamic nuclei. These thalamic relays establish reciprocal circuits with the M1, forming a closed-loop circuit that integrates motor commands and nociceptive processing (53). Optogenetic stimulation of Crus I also modulates M1 via the dentate nucleus, influencing whisker movement and tactile responses in rodents and highlighting its role in sensorimotor integration (54). This circuitry is depicted in Figure 4.

The functional coupling between the cerebellum and M1 is particularly relevant for pain modulation, given that M1 stimulation induces analgesic effects in chronic pain conditions. Such effects are attributed to the antidromic recruitment of thalamocortical circuits and engagement of descending modulatory systems (22, 64–66). Moreover, cerebellar output pathways appear to influence not only sensorimotor dimensions but also affective–motivational components of pain, involving key limbic regions such as the ACC and anterior insula (22, 42, 67).

Beyond M1, cerebellar projections from lobules IV–VI and Crus I also target medial prefrontal areas. Specifically, lobules IV–V project to the ACC, while vermal lobule VI and Crus I are connected to the prelimbic and orbitofrontal cortices (68, 69). The medial prefrontal cortex is involved in emotion regulation, memory-guided decision-making, and higher-order behavioral control (70, 71). Its role in pain modulation—particularly in descending control, ruminative processing, and comorbid affective symptoms such as anxiety and depression—is well supported by both preclinical and clinical studies (72–74). Taken together, this evidence supports a role for cerebellar output in modulating both sensory-discriminative and cognitive-affective aspects of pain.

Pain- and motivation-related brain regions, such as the nucleus accumbens (NAc) and the VTA, can be modulated by cerebellar output. While direct anatomical projections from the cerebellum to the NAc have not been conclusively identified, studies of functional connectivity and evidence for polysynaptic relay circuits suggest that cerebellar activity can influence NAc function. In contrast, the modulatory pathway from Purkinje cells in Crus I to VTA neurons—mediated via the dentate nucleus—is more clearly established (68, 75). Both the NAc and VTA are key components of mesolimbic circuits responsible for encoding the salience and motivational valence of pain. Consistently, chemogenetic activation of Crus I Purkinje cells projecting to the VTA has been shown to attenuate depressive-like behaviors in chronically stressed mice, reinforcing the notion that cerebellar circuits contribute to the regulation of affective states and mood-related comorbidities commonly observed in chronic pain (75).

The cerebellum also communicates with the hippocampus, a region central to memory and contextual processing of pain. Vermal lobule VI and Crus I influence the dentate gyrus via fastigial and dentate nuclei through polysynaptic thalamic–septal relays (76). Optogenetic stimulation of vermal lobules IV–V and hemispheric lobule VI modulates hippocampal activity and alters performance on hippocampus-dependent spatial tasks (77). The hippocampus itself exhibits structural and functional abnormalities in both rodent models of neuropathic pain and patients with chronic low back pain and complex regional pain syndrome (78). Chronic pain impairs the extinction of contextual—but not cued—fear memories, indicating a selective disruption of hippocampus-dependent learning, associated with decreased neurogenesis and synaptic remodeling. These findings suggest that cerebellar modulation of hippocampal circuits may contribute to maladaptive emotional and mnemonic responses observed in persistent pain states.

## 3 Transcranial direct current stimulation -technical factors

### 3.1 Methods

The methodology of this systematic review followed the PRISMA (Preferred Reporting Items for Systematic Reviews and Meta-Analyses) guidelines. No protocol was registered or published in PROSPERO (International Prospective Register of Systematic Reviews) before the development of this study.

Relevant studies were identified through comprehensive searches of the Cochrane Library (from 1996), PubMed (from 1996), and Embase (from 1993). The MeSH terms and entry terms used, along with their combinations, were: [(“Cerebellum”) AND (“Pain” OR “tDCS”) AND (“tDCS”)] (search conducted up to December 11, 2024). Additionally, the reference lists of the included studies were manually reviewed to identify other potentially eligible articles.

### 3.2 Study selection and eligibility criteria

To be eligible, studies were required to meet the following inclusion criteria: (1) involve human participants; (2) be published in English, Portuguese, or Spanish. In addition to English, Portuguese and Spanish were included because all reviewers are fluent in these languages, which ensured accurate assessment and data extraction without introducing interpretation bias; and (3) be classified as clinical studies, randomized controlled trials, systematic reviews, meta-analyses, or book chapters. Eligible studies specifically investigated the application of ctDCS for pain modulation, either in individuals with clinical pain or in healthy participants undergoing experimental pain induction. No restrictions were placed on the year of publication.

Title and abstract screening were conducted independently by two reviewers. In cases of disagreement, a third reviewer was consulted to reach a consensus. Duplicate records were removed before screening. Studies involving animal models or not aligned with the review objectives were then excluded.

The initial selection targeted studies with titles containing key terms such as “pain” and “cerebellum,” “pain” and “cerebellar tDCS,” or simply “tDCS.” Articles using synonymous or related MeSH terms were also considered. Abstracts were reviewed to assess relevance to ctDCS as a therapeutic intervention for pain or to the cerebellum’s role in nociceptive processing. Full-text articles were then analyzed, and only those that fulfilled all inclusion criteria were retained.

For each included study, the following data were extracted: first author, year of publication, study design, number of participants, electrode montage and stimulation site, stimulation parameters, reported adverse events (if any), neurophysiological outcomes (when applicable), pain assessment methods, study objectives, and main findings.

### 3.3 Quality and bias risk assessment

The methodological quality of the included studies was independently assessed by two reviewers using the Cochrane Risk of Bias Tool for Randomized Trials (RoB 2). In cases of disagreement, a third reviewer was consulted to reach a consensus. The domains evaluated included the randomization process, potential bias arising from period and carryover effects (when applicable), deviations from intended interventions, missing outcome data, outcome measurement, and selection of the reported results.

Following the assessment, the overall risk of bias for each study was categorized as “low risk,” “some concerns,” or “high risk.” It is important to note that the specific domains assessed varied according to the study design, such as parallel group randomized controlled trials or randomized crossover trials.

## 4 Results

**Figure 5:**
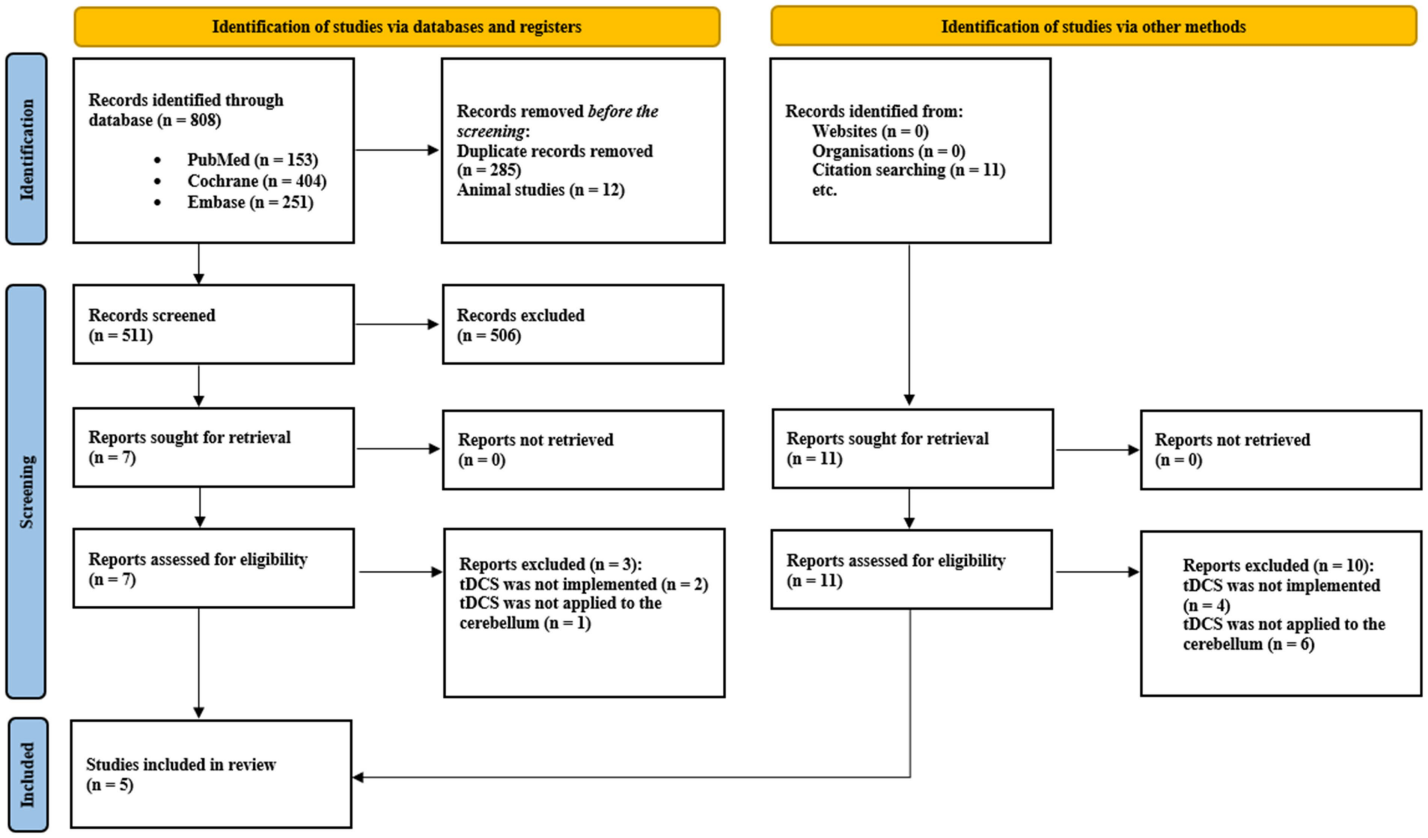
PRISMA fowchart of the included studies.

A summary of the search strategy is illustrated in Figure 5, following the PRISMA flowchart methodology. A total of 819 records were initially identified—808 through database searches and 11 through manual screening of bibliographic references. After removing duplicates and excluding studies involving animal models, 511 records remained for title and abstract screening. Studies that did not address the use of tDCS for pain management or the relationship between the cerebellum and pain processing were excluded. Subsequently, 18 full-text articles were assessed for eligibility, of which only five met the predefined inclusion criteria. These five studies specifically investigated the effects of ctDCS on pain modulation, either in healthy participants exposed to nociceptive stimuli or in patients with pain-related conditions.

### 4.1 Demographic characteristics of the patients included

The five randomized clinical trials included a total of 80 participants. Of these, 66 were healthy individuals enrolled across four studies, while 14 were patients with unilateral upper limb amputation (eight with left-sided and six with right-sided amputation), assessed in a single study (17). Four studies employed a crossover randomized controlled trial (RCT) design (15, 17–19), and one utilized a parallel-arm RCT design, including 16 healthy participants as controls (16). All participants in the active intervention arms were adults, with a mean age of approximately 28.5 years (SD = 7.0). A total of 47 women were included, representing a slight difference compared to men across both studies involving healthy individuals and those involving participants with chronic pain.

### 4.2 Stimulation protocol

The stimulation protocols employed across the studies typically positioned the target electrode (anode, cathode, or sham) along the midline, 2 cm below the inion, with the lateral edges placed approximately 1 cm medial to the mastoid processes. The placement of the reference electrode varied among studies: in three investigations, it was positioned on the right shoulder (16, 17, 19); in the remaining two, it was placed on the lateral aspect of the upper arm near the deltoid region (18) and the right buccinator area (15), respectively. The electrodes used had surface areas of either 35 cm^2^ (17, 18) or 25 cm^2^ (15, 16, 19).

All studies applied a stimulation intensity of 2 mA, and sham stimulation being discontinued after the ramp-up phase. Session duration and frequency varied. Four studies delivered a single session, whereas one study (17) implemented five consecutive daily sessions. Regarding duration, three studies (17–19) used 20-min sessions; one used 15-min (16) and one 5-min (15) protocols.

### 4.3 Neurophysiological tools and pain outcome measures

In three studies (16, 17, 19), laser stimulation combined with EEG was employed to assess perceptual thresholds, pain perception, and laser-evoked potentials (LEPs). Additionally, two studies incorporated peripheral electrical stimulation with surface electrodes into their protocols. Pereira et al. used cutaneous stimulation to quantify sensory and pain thresholds (15), whereas Stacheneder et al. (18) applied electrical sural-nerve stimulation and EEG to record spinal and cortical responses, as well as subjective pain ratings. In the same study, thermal stimuli and the conditioned pain modulation (CPM) paradigm were applied to evaluate endogenous pain modulation and to determine individual heat pain thresholds (18).

Subjective pain intensity was assessed using two different scales: the Visual Analog Scale (VAS) was employed in three studies (15, 17, 19), while the Numerical Rating Scale (NRS) was used in two studies (16, 18).

### 4.4 Adverse effects

In general, the studies included in this review did not report any adverse effects associated with ctDCS. An exception was observed in the study by Stacheneder et al., which described mild and transient side effects, including itching at the electrode site and post-session headaches (18).

### 4.5 Overview of study outcomes

The evidence gathered from the reviewed studies consistently supports the efficacy of ctDCS in modulating pain processing in both healthy individuals and patients with chronic pain conditions. Three studies reported polarity-dependent effects of ctDCS (16, 18, 19). Anodal stimulation was associated with increased pain thresholds, reduced scores on the VAS, and enhanced endogenous pain inhibition. In contrast, cathodal stimulation tended to decrease pain thresholds and impair inhibitory responses. Neurophysiological data supported these behavioral findings. Anodal ctDCS resulted in reduced amplitudes and prolonged latencies of the N1 and N2/P2 components in EEG recordings, whereas cathodal stimulation led to increased amplitudes and shortened latencies of the same components.

Additional support for the analgesic potential of anodal ctDCS was provided by Bocci et al. (17) and Pereira et al. (15). In patients with phantom limb pain, anodal stimulation reduced the frequency of paroxysmal pain episodes and alleviated non-painful phantom sensations (17). In healthy participants, it consistently increased pain thresholds, suggesting a facilitative effect on endogenous pain modulation mechanisms (15). Table 1 summarizes the demographic characteristics of the participants, the study designs, the main specifications of the stimulation protocols, and the reported outcomes across the included studies.

**Table 1:** Demographic characteristics and protocol specifications (*n* = 80).

Reference	Study design	Active group (*n*)	Sham group (*n*)	Anode location	Cathode location	tDCS protocol	Adverse effects	Neurophysiological measurements	Pain outcome used	Notes (aim, results)
Bocci et al. (19)	RCT, crossover design	15 (7/8 M)	In a crossover study, participants underwent three interventions—anodal, cathodal, and sham stimulation—with 1- week interval between sessions	For anodal stimulation, the electrode was centered on the median line, 2 cm below the inion, with its lateral borders positioned 1 cm medially to the mastoid apophysis. For cathodal stimulation, the electrode was placed on the right shoulder	For cathodal stimulation, the electrode was centered on the median line, 2 cm below the inion, with its lateral borders positioned 1 cm medially to the mastoid apophysis. For anodal stimulation, the electrode was placed on the right shoulder	2 mA, 20 min, 1 session of each stimulation with intervals of 1 week Cathodal, 2 mA, 20 min Anodal, 2 mA, 20 min Sham, current on only for the initial 30 s	Not informed	Laser stimulation was applied to the dorsum of the left hand. The PTh was quantified, and nociceptive laser stimuli were applied at two intensities (VAS1 and VAS2). N1 and N2/P2 components were captured using EEG RMT was assessed pre- and post-intervention using TMS	VAS	The study aimed to evaluate the effect of ctDCS on perceptive threshold, pain intensity, and laser-evoked potentials Result: Cathodal stimulation decreased the perceptive threshold, increased the VAS score, enhanced N1 and N2/P2 amplitudes, and reduced their latencies, while anodal stimulation had the opposite effect. Motor thresholds were not affected by the intervention
Bocci et al. (16)	RCT	16 highly hypnotizable volunteers (9/7 M) were recruited	There was no sham group; the control group consisted of 16 healthy subjects matched for age and gender, not selected based on hypnotizability.	For anodal stimulation, the electrode was centered on the median line, 2 cm below the inion, with its lateral borders positioned 1 cm medially to the mastoid apophysis For cathodal stimulation, the electrode was placed on the right shoulder	For cathodal stimulation, the electrode was centered on the median line, 2 cm below the inion, with its lateral borders positioned 1 cm medially to the mastoid apophysis For anodal stimulation, the electrode was placed on the right shoulder	2 mA, 15 min, 1 session of each stimulation was applied Cathodal, 2 mA, 15 min Anodal, 2 mA, 15 min	Not informed	Laser stimulation was applied to the dorsum of the left hand. The PTh was quantified, followed by eight nociceptive laser stimuli, with subjects rating the perceived pain. N1 and N2/P2 components were recorded using EEG	NRS	The study aimed to evaluate the effect of ctDCS on pain perception and LEPs in highly hypnotizable individuals compared to controls Result: In highly hypnotizable individuals, anodal stimulation increased N2/P2 amplitude without affecting perceived pain, while cathodal stimulation had no significant effects. In contrast, control participants reported decreased pain and reduced N1 and N2/P2 amplitudes after anodal stimulation, with the opposite pattern observed following cathodal stimulation.
Pereira et al. (15)	RCT, crossover design	14 (7/7 M)	In a crossover study, participants underwent three interventions—anodal, cathodal, and sham stimulation—with at least 5 h interval between sessions	For anodal stimulation, the electrode was centered on the median line, 2 cm below the inion, with its lateral borders positioned 1 cm medially to the mastoid apophysis For cathodal stimulation, the electrode was placed over the right buccinator area	For cathodal stimulation, the electrode was centered on the median line, 2 cm below the inion, with its lateral borders positioned 1 cm medially to the mastoid apophysis For anodal stimulation, the electrode was placed over the right buccinator area	2 mA, 5 min, 1 session of each stimulation with intervals separated by at least 5 h. Cathodal, 2 mA, 5 min Anodal, 2 mA, 5 min Sham, turned off after the ramp-up phase	Not informed	Electrical stimulation was applied to the proximal third of the right medial tibia. The ST was defined as a slight sensation in the leg (VAS 1), and the PThres as barely painful (VAS 4)	VAS	The study aimed to evaluate the effect of ctDCS on lower extremity sensory and pain thresholds. Result: Anodal stimulation increased the pain threshold, while cathodal and sham stimulations had no significant effect. No changes in sensory threshold were observed with any stimulation.
Bocci et al. (19)	RCT, crossover design	14 (8/6 M) with unilateral upper limb amputation	In a crossover study, participants underwent two interventions—anodal, and cathodal stimulation—with a 3-month interval between sessions	For anodal stimulation, the electrode was centered on the median line, 2 cm below the inion, with its lateral borders positioned 1 cm medially to the mastoid apophysis For cathodal stimulation, the electrode was placed on the right shoulder	For cathodal stimulation, the electrode was centered on the median line, 2 cm below the inion, with its lateral borders positioned 1 cm medially to the mastoid apophysis For anodal stimulation, the electrode was placed on the right shoulder	2 mA, 20-min sessions were applied to each type of stimulation—anodal, and cathodal—over five consecutive days, with three-month intervals separating each stimulation type. Cathodal, 2 mA, 20 min Anodal, 2 mA, 20 min	Not informed	Laser stimulation was applied to the stump to determine the PT, followed by twenty nociceptive laser stimuli, during which subjects rated their perceived pain. EEG was used to record the N1 and N2/P2 components Clinical evaluations were conducted at baseline (T0), at the end of the ctDCS week (T1), and at 2 weeks (T2) and 4 weeks (T3) post-intervention. The variables assessed included PLP intensity, pain paroxysms, stump pain, non-painful phantom limb sensations, and phantom limb movements	VAS	The study aimed to evaluate the effect of ctDCS on modulating nociceptive processing and pain perception in patients with painful and non-painful phantom limb sensations Result: Anodal ctDCS reduced paroxysmal pain, non-painful phantom limb sensations, and phantom limb movements
Stacheneder et al. (18)	RCT, crossover design	21 (16/6 M)	In a crossover study, participants underwent three interventions—anodal, cathodal, and sham stimulation—with a 5-day interval between sessions	For anodal stimulation, the electrode was centered on the median line, 1-2 cm below the inion For cathodal stimulation, the electrode was placed on the lateral upper arm	For cathodal stimulation, the electrode was centered on the median line, 1- 2 cm below the inion For anodal stimulation, the electrode was placed on the lateral upper arm	2 mA, 20 min, 1 session of each stimulation with intervals of 1 week Cathodal, 2 mA, 20 min Anodal, 2 mA, 20 min Sham, current on only for the initial 15 s	Itching under the electrode and headache following the procedure	The study recorded spinal and cortical responses to sural nerve stimulation, assessing the RIII reflex from the biceps femoris and SEPs from the vertex referenced to the forehead. Each 2-min cycle included 12 stimuli, and pain intensity was rated using an NRS after each cycle. Thermal stimuli were employed to assess pain perception and modulation mechanisms. This protocol determined heat pain intensity and offset analgesia CPM was evaluated using a test stimulus (a 30-s heat application) and a conditioning stimulus (a 60-s cold water immersion)	NRS	The study aimed to assess the impact of ctDCS on nociceptive processing and endogenous pain modulation. Result: Findings indicated that cathodal ctDCS increased pain perception and reduced endogenous pain inhibition, while anodal ctDCS enhanced endogenous pain inhibition

RCT Randomized Clinical Trial, PTh Perceptive Threshold, EEG Electroencephalogram, VAS Visual Analogue Scale, RMT Resting Motor Threshold, TMS Transcranial Magnetic Stimulation, ctDCS Cerebellar Transcranial Direct Current Stimulation, NRS Numerical Rating Scale, LEPs Laser Evoked Potentials, ST Sensory Threshold, PThres Pain Threshold, PLP Phantom Limb Pain, SEPs Somatosensory Evoked Potentials, CPM Conditioned Pain Modulation.

### 4.6 Bias risk assessment

**Figure 6:**
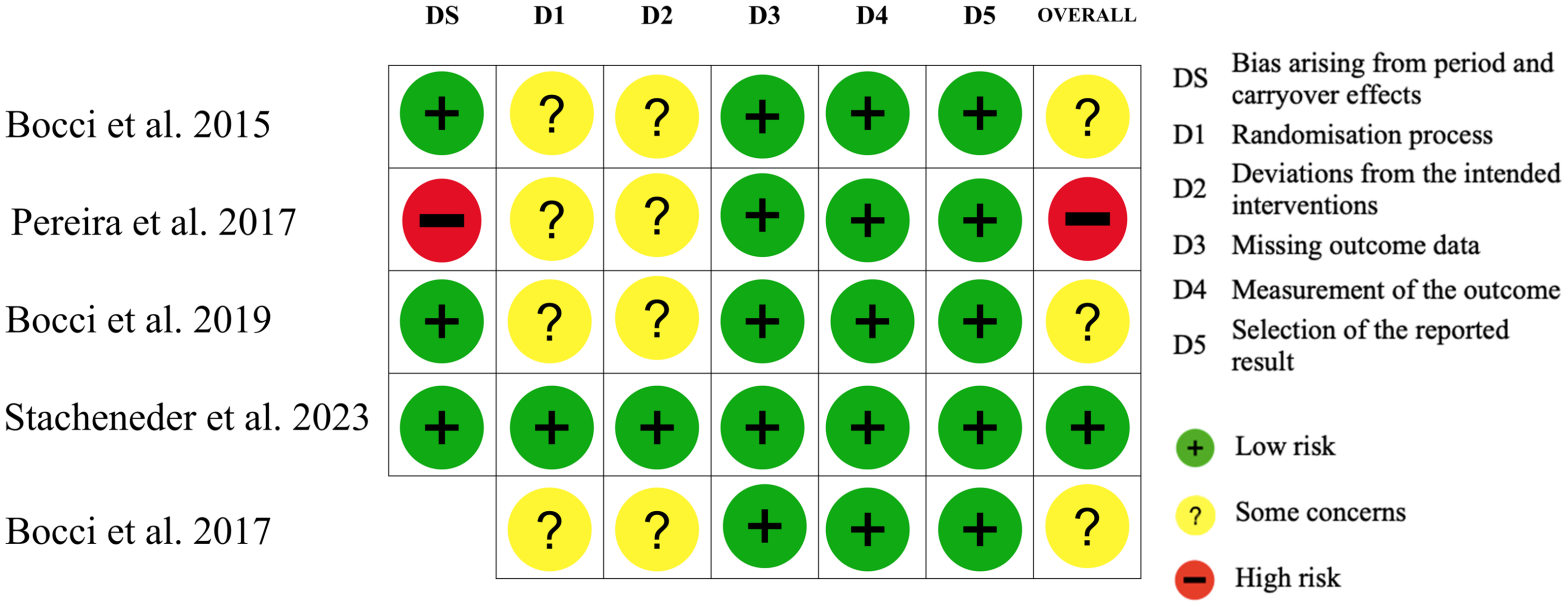
Risk of bias of studies included. Crossover trials (including the DS variable) and randomized clinical trials were conducted according to the authors’ interpretations and methodologies.

**Figure 7:**
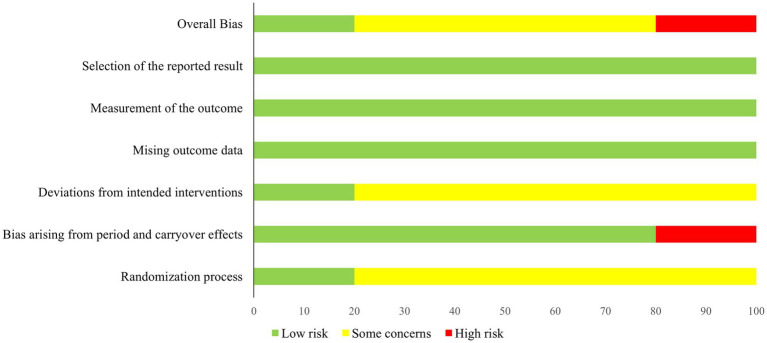
Risk of bias—authors’ judgments on each risk of bias item are presented as percentages across all included trials, assessed using the Cochrane risk of bias tool.

The risk of bias was assessed using the RoB 2 tool, as recommended by the Cochrane Collaboration, and is illustrated in Figures 6, 7. Overall, the main concerns identified across the studies included insufficient reporting of the randomization process (Domain D1 of RoB 2) and inadequate information regarding blinding of outcome assessors (Domain D2). Although not classified as a high risk, the studies by Pereira et al. (15) and Bocci et al. (19) lacked important details—such as demographic and clinical characteristics of the participants—that would have allowed for a more comprehensive characterization of the intervention groups.

In the study by Pereira et al. (15), a high risk of bias was identified due to potential carryover effects (Domain DS of RoB 2). Although the interval between sessions may vary among studies, conducting multiple sessions on the same day can lead to cumulative effects, which may confound the outcomes (15).

## 5 Discussion

Chronic pain remains a major clinical and societal burden, often refractory to conventional treatments and associated with significant adverse effects. In this context, ctDCS has emerged as a promising neuromodulatory technique. This systematic review provides an integrative synthesis of the stimulation protocols employed, the clinical characteristics of the populations studied, and the potential therapeutic effects of ctDCS in pain management. Despite preliminary findings suggesting polarity-dependent analgesic effects, the overall evidence remains limited, highlighting the need for more rigorous and standardized investigations.

### 5.1 Cerebellar tDCS and pain perception: mechanisms and effects

Evidence from the studies included in this review supports the role of ctDCS in modulating nociceptive processing through polarity-dependent mechanisms. In healthy volunteers, anodal stimulation consistently increased pain thresholds and reduced pain-related evoked potentials, whereas cathodal stimulation either enhanced pain sensitivity or produced no change (15, 16, 19). In phantom limb pain, anodal ctDCS reduced paroxysmal pain and phantom sensations (16), while Stacheneder et al. (18) showed that anodal stimulation enhanced inhibitory control and cathodal stimulation impaired descending inhibition, reinforcing polarity-dependent effects.

Mechanistically, it has been proposed that nociceptive inputs may converge on Purkinje cells, which exert inhibitory control over the dentate nucleus. Through these circuits, the cerebellum can influence thalamic and brainstem nociceptive relays that project to cortical regions involved in pain processing (12, 28, 42). The analgesic effects of ctDCS may rely on modulation of the cerebello–thalamo–cortical pathway, whose inhibitory influence has been consistently demonstrated via TMS-based cerebellar–brain inhibition (CBI) (79). Supporting this, Galea et al. (80) showed that anodal ctDCS increases Purkinje cell excitability, thereby reinforcing the inhibitory influence over the dentate nucleus and reducing thalamocortical drive. In contrast, cathodal stimulation produces the opposite effect, confirming the polarity-specific nature of cerebellar neuromodulation (80). Such bidirectional control becomes particularly relevant in chronic pain, where thalamic plasticity alters connectivity, excitability, and rhythmicity, leading to thalamocortical dysrhythmia that amplifies nociceptive salience and sustains persistent pain (81, 82). It is therefore plausible that ctDCS restores inhibitory control within the dentate–thalamo–cortical pathway, counteracting pathological oscillatory activity. This framework parallels the rationale in dystonia, where reduced cerebellar inhibition justifies anodal stimulation (83), and in tremor disorders, where abnormal olivocerebellar rhythmicity supports the use of cathodal interventions (84).

A complementary mechanism involves the anti-inflammatory and neuroprotective actions of tDCS. In experimental neuropathic pain models, repeated stimulation has been shown to downregulate pro-inflammatory cytokines (e.g., spinal IL-1β, hippocampal TNF-*α*), upregulate IL-10, and suppress microglial and astrocytic activation, thereby fostering adaptive plasticity and reducing hypersensitivity (85, 86). Although clinical evidence remains limited, these preclinical findings, when integrated with the polarity-dependent modulation of cerebellar circuits, suggest a dual potential of ctDCS: rebalancing dysfunctional thalamocortical activity and attenuating neuroinflammatory processes. Collectively, these mechanisms strengthen the conceptualization of the cerebellum as a central modulatory hub in pain pathophysiology and support ctDCS as a mechanism-based strategy for chronic pain management.

### 5.2 Determinants of interindividual variability in tDCS response

Interindividual variability in response to ctDCS is a critical challenge that limits the generalizability of its clinical effects. Beyond the stimulation parameters, factors such as neurochemical balance, inflammatory states, and genetic predispositions can substantially modulate outcomes, shaping both the magnitude and direction of neuromodulatory effects.

At the neurochemical level, anodal stimulation can reduce cortical GABA concentrations, as shown by spectroscopy (87). Its after-effects are also sensitive to the dopaminergic state, with evidence that receptor function/availability modulates the magnitude and direction of plasticity (88–90). Moreover, tDCS-induced plasticity critically depends on NMDA receptor mechanisms: antagonists abolish and partial agonists enhance these effects, indicating that departures from an optimal glutamatergic balance can dampen these neurophysiological changes (91, 92). In neuropathic conditions, reduced GABAergic tone and disruption of opioid signaling further compromise responsiveness (93). Consistent with this, PET imaging demonstrated that anodal tDCS over M1 enhances endogenous opioid release and increases *μ*-opioid receptor binding, highlighting the role of opioid-mediated mechanisms to analgesia (94, 95). Neuroinflammation also emerges as a critical determinant: elevated cytokines such as TNF-*α*, IL-1β, and IL-6 destabilize synaptic homeostasis, impair inhibitory neurotransmission within descending pain pathways, and exacerbate central sensitization, ultimately limiting the therapeutic impact of tDCS (96–98). Genetic polymorphisms further contribute to variability. The BDNF Val66Met variant affects activity-dependent plasticity and functional connectivity, influencing both pain modulation and responsiveness to stimulation (99, 100). Similarly, COMT Val158Met, which alters dopaminergic tone, has been linked to differences in cognitive flexibility and neuromodulatory outcomes (101–103).

In sum, ctDCS responsiveness appears intrinsically state-dependent: the neurochemical milieu, inflammatory activity, and genetic background gate both the sign and magnitude of induced plasticity, offering a coherent explanation for the between- and within-subject heterogeneity observed to date.

### 5.3 Clinical characteristics of the study population

In the present review, most included ctDCS studies were conducted in healthy young adults, which restricts the applicability of their findings to clinical pain populations. Only one trial specifically investigated patients—upper-limb amputees with phantom limb pain—reporting that anodal ctDCS reduced paroxysmal pain and phantom sensations (17). Among studies with healthy participants, polarity-dependent effects on pain perception were observed only in low-hypnotizable individuals, suggesting that cognitive traits may modulate cerebellar–cortical interactions and shape the neuromodulatory impact of ctDCS (16).

Demographic variables are also relevant. The predominance of young female samples introduces potential bias, as sex-related anatomical factors—such as greater skull thickness in men—may reduce current penetration (104). In addition, age-related gray matter atrophy and increased cerebrospinal fluid volume can alter current flow and target engagement (105). Hormonal fluctuations—particularly in estrogen and cortisol—may further modulate excitability and plasticity, adding variance to ctDCS outcomes (106). Medications and commonly used substances also represent important confounders. Anticonvulsants, benzodiazepines, and other GABAergic agents may attenuate stimulation-induced plasticity, whereas SSRIs may prolong facilitatory after-effects (107–109). Caffeine and nicotine have also been shown to modulate cortical excitability in a dose-dependent manner (110, 111).

Viewed across studies, sample composition is a first-order determinant of observed effects: clinical diagnosis, cognitive traits, sex/age/hormonal status, and medication/substance exposure shape current delivery and plasticity, and thereby the size and stability of ctDCS outcomes.

### 5.4 Anatomical and technical determinants of ctDCS efficacy

Despite the use of standardized protocols—typically positioning the active electrode 1–2 cm below the inion and 1 cm medial to the mastoids to target the cerebellar vermis—reported outcomes remain inconsistent: some studies demonstrate robust neuromodulatory effects, whereas others do not detect significant changes. One major factor contributing to this variability is interindividual anatomical diversity, which shapes both the magnitude and spatial distribution of the electric field within cerebellar structures. Computational modeling studies have shown that the distance between the scalp and cerebellum is the most influential anatomical variable, accounting for up to 60% of the variance in intracerebellar electric field strength (112–114). Additional morphometric features—such as the angulation of the cerebellar and pontine fossae—can further shape the trajectory and depth of current penetration (115). These anatomical variations imply that even under standardized electrode placement, the resulting neural engagement can differ substantially across individuals. Emerging evidence suggests that variability in local electric field strength has functional consequences. Studies have reported that differences in field intensity are associated with changes in GABA concentrations, modulation of motor-evoked potentials, and alterations in cerebello-cortical connectivity (90, 116–118). Together, these findings indicate that anatomical features not only affect current delivery but also determine the neurophysiological and behavioral outcomes of ctDCS, underscoring the importance of individualized targeting strategies.

In addition to anatomical variability, the electrode montage plays a decisive role in shaping current distribution. Cephalic montages, in which the reference electrode is placed over frontal or supraorbital regions, can generate stronger overall fields but with greater spatial dispersion, potentially engaging adjacent cortical structures such as the prefrontal and occipital cortices (112, 119). In contrast, extracephalic montages—where the reference electrode is positioned on the shoulder or buccinator muscle—tend to produce more focal stimulation of cerebellar targets while reducing unintended spread to supratentorial regions (114, 120). Despite these well-documented biophysical differences, current evidence does not clearly demonstrate whether one montage translates into superior clinical efficacy.

Stimulation parameters are another critical determinant of ctDCS efficacy. Most protocols apply 2 mA for 15–25 min across multiple sessions interspersed with rest days, a regimen considered the current standard. Shorter applications, such as 5 min, can induce transient changes in cortical excitability (9, 121), but their ability to produce sustained modulation of cerebellar circuits remains uncertain. To ensure effective engagement, Habas et al. recommend using at least 1.5 mA (36), whereas Workman et al. (122) demonstrated that 4 mA is safe and well tolerated in patients with Parkinson’s disease. Importantly, posterior-fossa morphometrics—such as scalp-to-cerebellum distance and the cerebellopontine angle—further determine how stimulation intensity translates into effective current delivery (114). The effects of 2 mA protocols, however, must be interpreted with caution. Several studies included in this review found that anodal ctDCS increased pain thresholds and reduced pain perception, whereas cathodal stimulation lowered thresholds and enhanced pain perception (16, 18, 19). Nonetheless, evidence from non-cerebellar paradigms highlights important inconsistencies. Batsikadze et al. (123) reported that cathodal tDCS over M1 at 2 mA paradoxically enhanced excitability, producing facilitatory effects similar to anodal stimulation. In line with this, Vimolratana et al. (124) showed that both anodal and cathodal stimulation at 2 mA increased muscle strength in healthy participants, while cathodal stimulation at 1–1.5 mA produced the expected inhibitory effects. Dyke et al. (125) added further nuance by showing that 2 mA stimulation yields highly variable outcomes: while anodal stimulation increased excitability at the group level, its effects were poorly reliable within individuals, and cathodal stimulation failed to produce consistent changes either within or between subjects. Overall, the evidence suggests that, outside the cerebellum, 2 mA stimulation may not reliably induce polarity-specific responses; instead, observed changes may reflect non-linear or state-dependent physiology rather than genuine polarity-dependent modulation. By contrast, in the cerebellum, polarity-dependent effects of ctDCS have been demonstrated: anodal tends to strengthen, whereas cathodal tends to reduce, the cerebellar inhibitory influence on cortical excitability (80). Importantly, any apparent polarity effects should be interpreted in the context of effective intracerebellar field strength, montage-dependent current distribution, and individual posterior fossa morphology, which together determine target engagement and downstream effects.

From a translational standpoint, montage selection and stimulation parameters should be aligned with the intended neuromodulatory goal. Future progress will depend on integrating high-resolution imaging, individualized electric-field modeling, and systematic evaluation of stimulation intensity and dosing schedules to consolidate polarity-sensitive interventions and establish the therapeutic potential of ctDCS for chronic pain.

## 6 Future directions

Recent evidence supports a paradigm shift in pain neuroscience, highlighting the cerebellum not merely as a motor structure but as a central hub that integrates sensory, affective, and cognitive dimensions of pain processing. In this context, ctDCS emerges as a promising technique to modulate distributed pain-related networks.

One important gap in the current literature is the limited use of inflammatory and neuroimmune biomarkers as outcome measures. Experimental studies in neuropathic pain models suggest that tDCS exerts anti-inflammatory and neuroprotective effects, including the down-regulation of pro-inflammatory cytokines (e.g., TNF-*α*, IL-1β), the up-regulation of IL-10, and the suppression of microglial and astrocytic activation, thereby fostering adaptive plasticity and reducing hypersensitivity (85, 86). Incorporating such biomarkers in clinical trials may provide a translational link between molecular pathways and behavioral outcomes, clarifying whether modulation of neuroinflammatory cascades contributes to the analgesic effects of tDCS, tACS, or rTMS in chronic, drug-resistant pain syndromes.

Another promising avenue is the use of multi-target stimulation protocols. Preliminary evidence suggests that concurrent anodal stimulation of M1 and the cerebellum can induce synergistic plasticity, producing greater facilitatory effects than single-site stimulation (126). Extending this rationale, combined cerebellar–spinal tDCS may enhance descending modulatory control. Advances in computational modeling and “deep NIBS” approaches also offer the possibility of predicting and optimizing electric field distribution within deep cerebellar nuclei and subcortical structures, providing novel, non-invasive ways to engage circuits traditionally considered inaccessible (127).

Finally, the integration of neurophysiological and imaging techniques (e.g., EEG, fMRI, TMS) remains essential to clarify the mechanisms of ctDCS and identify biomarkers predictive of treatment response. Such multimodal approaches will not only improve mechanistic understanding but also guide the development of personalized and network-oriented stimulation strategies.

Overall, future research should prioritize biomarker-based validation, multi-target stimulation designs, and multimodal neurophysiological assessments. This integrative approach may enhance reproducibility, reduce variability, and consolidate ctDCS as a precision tool for mechanism-based interventions in chronic pain.

## 7 Limitations

When interpreting the findings of this review, several limitations must be acknowledged. First, most studies included small sample sizes predominantly composed of healthy young female participants, which limits statistical power and reduces the generalizability of results to clinical populations. Second, the widespread use of single-session ctDCS protocols prevents meaningful conclusions about cumulative or long-term effects. As tDCS-induced plasticity relies on activity-dependent mechanisms, both the number and frequency of sessions are likely to influence clinical outcomes. Therefore, longitudinal studies involving repeated ctDCS applications are needed to evaluate the durability and therapeutic relevance of its effects over time.

Third, another important limitation is the absence of advanced neuroimaging techniques—such as functional near-infrared spectroscopy and functional magnetic resonance imaging—which hinders mechanistic interpretation. Without such tools, it remains unclear whether observed changes in pain perception result from direct cerebellar modulation or downstream effects on broader cortical networks. Integrating multimodal neuroimaging in future research could enhance mechanistic understanding and support the development of more targeted and effective interventions.

Fourth, a further limitation relates to the prevalent use of 2 mA stimulation. Evidence from M1 tDCS indicates that cathodal stimulation at 2 mA may fail to produce the canonical inhibitory effect and can even yield facilitatory changes; although this has not been demonstrated directly for ctDCS, several studies in this review using 2 mA have reported polarity-dependent analgesic effects. Given target-specific differences between M1 and the cerebellum, interpretations of cathodal ctDCS at 2 mA should be made with caution. Future trials should include systematic dose–response comparisons (e.g., 1–1.5 mA vs. 2–4 mA), immediate and delayed outcome assessments, and repeated-session designs to clarify intensity–polarity interactions.

Fifth, heterogeneity in stimulation parameters—spanning electrode montage, current polarity, session duration, and outcome measures—complicates cross-study comparisons. Standardized reporting and methodological harmonization are essential for improved reproducibility and the identification of optimal stimulation protocols.

Sixth, only a small number of studies included clinical populations, and those that did focused primarily on individuals with phantom limb pain—a condition characterized predominantly by neuropathic mechanisms. As a result, it remains uncertain whether similar effects would be observed in other pain phenotypes, such as nociplastic pain (e.g., fibromyalgia), nociceptive pain (e.g., osteoarthritis), or other neuropathic conditions (e.g., diabetic neuropathy). These conditions differ in their underlying neurobiology, including degrees of central sensitization, descending pain inhibition, and ongoing peripheral input, all of which may influence responsiveness to ctDCS. Future studies should explicitly address these pain subtypes to clarify the broader applicability of cerebellar stimulation.

Seventh and lastly, the influence of individual psychological and biological characteristics—such as anxiety, pain catastrophizing, pain sensitivity, and placebo responsiveness—was rarely considered in the reviewed studies. Incorporating psychometric and biological markers in future protocols will be essential for elucidating interindividual variability in response to ctDCS and for guiding the development of personalized neuromodulatory interventions.

## 8 Conclusion

The ctDCS has emerged as a promising non-invasive technique for modulating nociceptive processing and may provide therapeutic benefits for individuals with chronic pain—even after a single session. However, its clinical efficacy is shaped by a complex interplay of anatomical, neurochemical, cognitive, and psychological factors that influence individual responsiveness to neuromodulation.

To enhance its translational potential, ctDCS should be integrated into personalized, network-based strategies, ideally in combination with stimulation of other cortical regions. Optimizing protocols—through tailored selection of stimulation parameters, electrode montage, and dosing schedules—requires mechanistic insight and careful consideration of patient-specific features. Incorporating neurophysiological and neuroimaging assessments within a biopsychosocial framework will be essential for refining interventions and identifying biomarkers of treatment response.

Collectively, the available evidence indicates that the effects of ctDCS in chronic pain are modulated by neurochemical, inflammatory, and genetic factors. Addressing these dimensions through precision neuromodulation approaches will be key to improving clinical outcomes and establishing ctDCS as a viable therapeutic tool in pain management.

## Data Availability

The datasets generated and/or analyzed during the current study are available from the corresponding author on reasonable request.
